# Global research trends on melasma: a bibliometric and visualized study from 2014 to 2023

**DOI:** 10.3389/fphar.2024.1421499

**Published:** 2024-07-25

**Authors:** Li-Jun Wang, Yao-Bin Pang, Wen-Quan Li, Qing-Ying He, Xue-Er Zhang, E. Liu, Jing Guo

**Affiliations:** ^1^ School of Clinical Medicine, Chengdu University of Traditional Chinese Medicine, Chengdu, China; ^2^ Department of Dermatology, Hospital of Chengdu University of Traditional Chinese Medicine, Chengdu, China

**Keywords:** melasma, melanosis, globle research trends, bibliometric, visualized study

## Abstract

Melasma, a prevalent pigmentary disorder, is characterized by its complex etiology, propensity for recurrence, and resistance to treatment. However, there is currently no research on melasma through bibliometrics and visualisation. This study analyses the hotspots and trends in the field based on 2,709 publications from the Web of Science Core Collection (WOSCC). We carried out bibliometric analyses using Citespace software for different countries/regions, institutions, authors, and keywords. References were also analysed using VoSviewer. The results indicate that overall, there has been an increase in publications related to melasma since 2014. According to the analysis of the collaborative network diagram, the United States, Egyptian Knowledge Bank, and Benjakul Soottawat are the most contributing countries, institutions, and authors, respectively. Reference and keyword analyses have identified the pathogenesis and treatment of melasma as a prevalent topic in recent years. And how to find new treatment options and more effective therapeutic drugs is a future research trend. This is the first bibliometric and visual analysis of melasma-related literature to explore research hotspots and trends.

## Introduction

Melasma is an acquired hyperpigmentation acquired disorder. To date, the treatment of melasma remains challenging. Management of melasma is also extremely important due to inconsistent results and frequent recurrences (Jo et al., 2024). Worldwide, the prevalence of melasma is as high as 41% in some regions (Handel et al., 2014). Furthermore, melasma predominantly targets the facial region, exerting a detrimental influence on patients’ overall wellbeing and directly damaging their psychological and emotional state (Handel et al., 2014). However, the mechanism of melasma is unknown. The traditional mechanism for this is an increase in melanin vesicles synthesized by melanocytes and abnormally clustered in certain areas. (Kwon et al., 2019). Although the exact cause is unknown, some explanations include inflammation, hormonal fluctuations in the body, hereditary predisposition, and UV radiation (Lee, 2015).

Current treatments for melasma include photoprotection, lasers, topical hydroquinone, corticosteroids, and chemical peels (Gan and Rodrigues, 2024). An evidence-based review article mentions that hydroquinone monotherapy or triple cream (hydroquinone, retinoid, and corticosteroid) is a relatively effective approach, whereas laser therapy may be less effective than topicals and carry a higher risk of adverse effects (McKesey et al., 2020). The efficacy and safety of picosecond lasers in treating melasma, as well as the choice of wavelength, need to be evaluated with a larger sample size (Feng et al., 2023). Rashmi Sarkar et al. mentioned that many of some of the newer medications for melasma are botanicals, which are safer compared to traditional medications (Sarkar et al., 2020).

A metrological analysis of the existing literature, focusing primarily on “Quality of randomized controlled trials of melasma treatments,” among others (Chen et al., 2015). However, global research trends in melasma have not been systematically studied through bibliometric and visual analyses. Bibliometric and visualisation analyses, grounded in the WOSCC, were conducted utilizing Citespace (Cortese et al., 2022) and Vosviewer (Mu et al., 2022). The aim of this study is to comprehensively and systematically review the current state of global research on melasma from 2014 to 2023 and to fill the gap in the bibliometric analyses of this research area.

## Materials and methods

### Data sources

**FIGURE 1:**
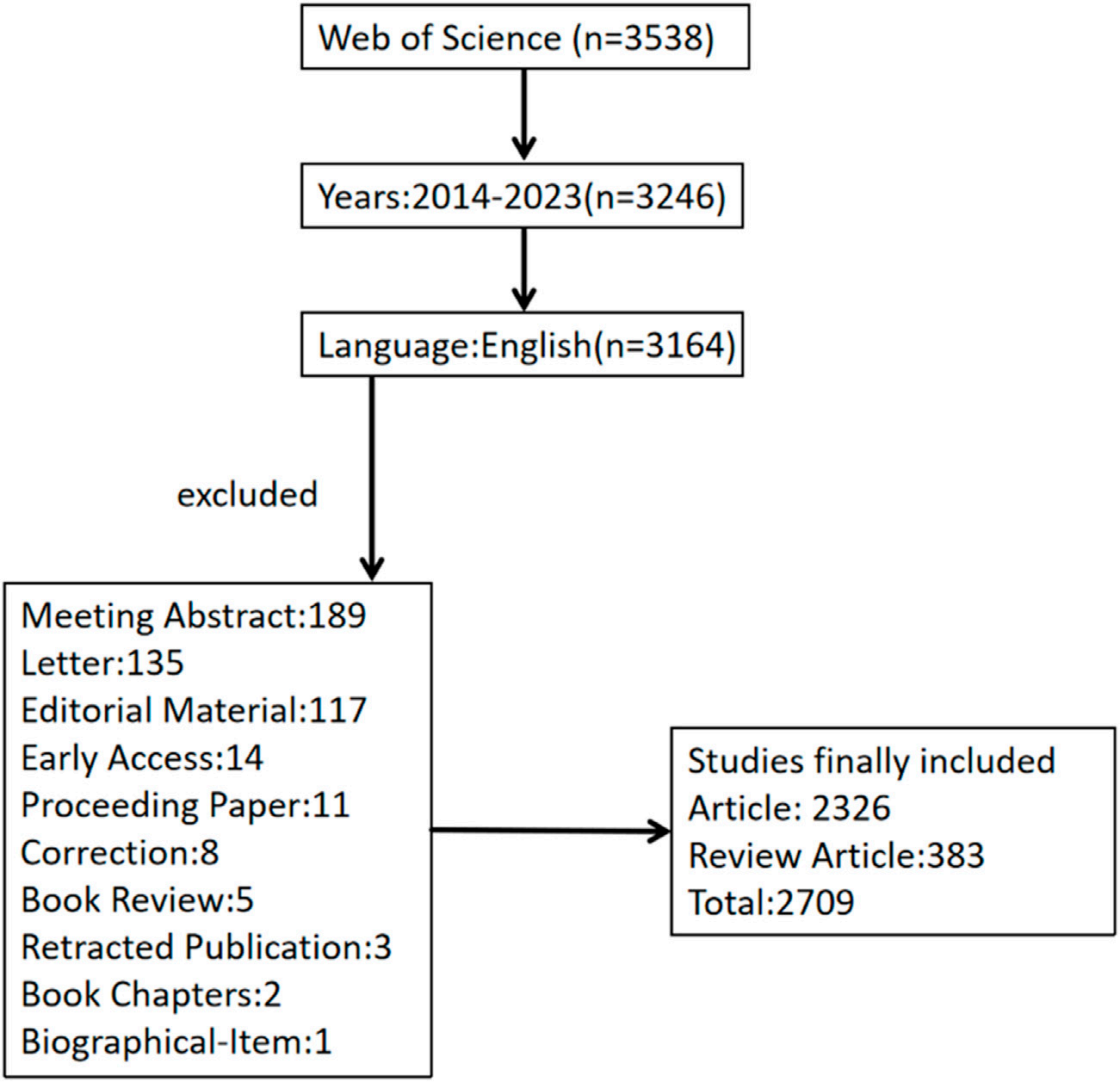
Flow diagram of the included articles.

We chose the Web of Science Core Collection (WOSCC) of Science Citation Index Expanded as the search database for this study. WOSCC is the largest and most comprehensive database covering the literature available. It is highly authoritative in scientific research and bibliometric studies. We used the search formula [TS = (Melanosis OR Melanoses OR Melanism OR Freckles OR Freckle OR Chloasma OR Chloasmas OR Melasma OR Melasmas)] for the retrieval. Set the publication date condition of the retrieved documents to “2013-01-01 to 2023-12-31.” We limit the search language to English and set the document type to “Article or Review.” The detailed search results are shown in Figure 1.

### Data analysis

The data analysis software used in this study was Citespace.6.2.R6, Vosviewer 1.6.20 and Microsoft Office Excel 2021. The specific parameters in Citespace are set as follows: time slicing: January 2014–December 2023, Term source: Title, Abstract, Author Keywords and Keywords Plus, Node type: Author, Institution, Country, Keyword. Link strength: cosine. Selection criteria: Top N = 50.

## Results

### Literature search results

We searched 3,538 related literatures through the Web of Science Core Collection (WOSCC) database and screened 2,709 articles. The specific filtering process is shown in Figure 1.

### Publication trend analysis

**FIGURE 2:**
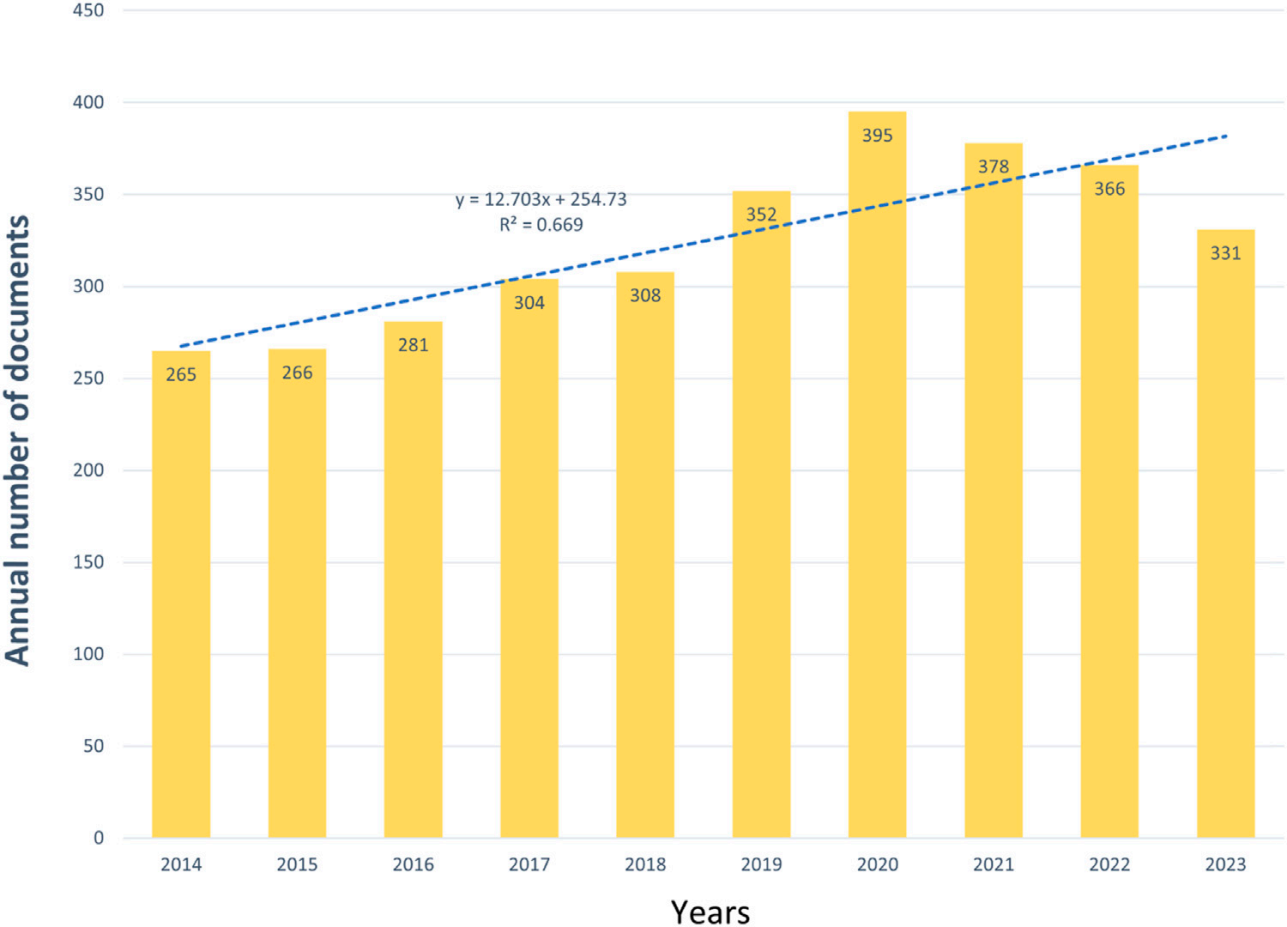
Published trend chart concerning melasma.

As shown in Figure 2, the number of published articles is at least 265 in 2014 and up to 395 in 2020, with a linear growth trend in the number of published articles, but a slight decrease in the number of published articles in 2021–2023. The decline may be due to the fact that melasma, as a subject that has been studied for a long time, may have reached a certain level of research saturation in some areas, leading to fewer new research breakthroughs. In summary, the field of melasma research has garnered increasing attention, warranting the pursuit of novel research breakthroughs since 2021.

**FIGURE 3:**
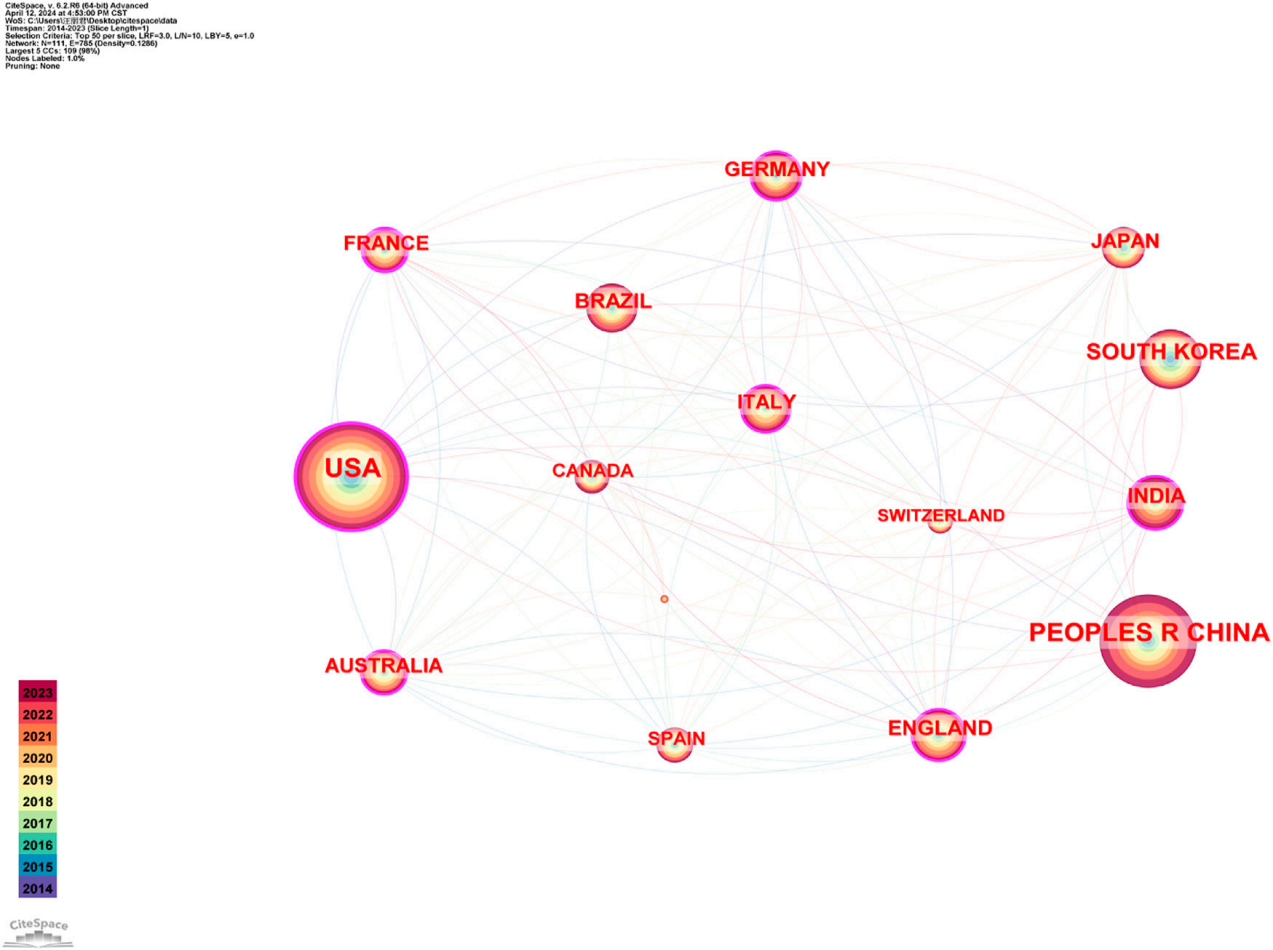
Country/region collaboration network of research on melasma.

We looked at the collaborative network of countries/regions, institutions and authors of research related to melasma. Table 1 presents all countries/areas in the top 10 based on number of publications and centrality value. Concurrently, the organizational network map (Figure 3) depicted 111 nodes and 785 link routes. The size of a node correlates positively with the number of articles published. The outer circle of the node is in purple to indicate that the centrality is greater than 0.1. The greater the value of centrality, the more the node cooperates with other nodes. Figure 3 illustrates that the United States leads in the publication of melasma research articles, with China and South Korea ranking second and third, respectively. This suggests the prominent research capabilities of these nations in the melasma research domain. The United States has the largest centrality value, suggesting that it has the most cooperation with countries. In China, 471 articles have been published on melasma research; however, the centrality value stands at a mere 0.06, indicating a potential deficiency in international collaboration within this research domain. Korea and Japan do not have a low article publication volume, but their centrality values are 0.04 and 0.08. All of this suggests that Asia needs to further strengthen international cooperation in the field of melanosis research.

**TABLE 1:** Countries/regions, institutions, authors ranked by publications and centrality.

Item	Ranking	Name	Publications	Name	Centrality
Country	1	United States	579	United States	0.40
	2	Peoples R China	471	France	0.20
	3	South korea	213	Australia	0.19
	4	India	161	Germany	0.17
	5	Brazil	151	India, England	0.12
	6	England	146	Italy	0.11
	7	Italy	131	Brazil, Japan	0.08
	8	Germany	128	Peoples R China	0.06
	9	Australia	115	South Korea, Canada, Switzerland, Jordan	0.04
	10	Japan	113	Spain	0.03
Institution	1	Egyptian Knowledge Bank	80	University of California System	0.27
	2	University of California System	61	Institut National de la Sante et de la Recherche Medicale	0.12
	3	Harvard University	50	Monash University	0.11
	4	Centre National de la Recherche Scientifique	34	Egyptian Knowledge Bank, Harvard University, Centre National de la Recherche Scientifique, Chinese Academy of Sciences	0.10
	5	Universidade Estadual Paulista	33	Seoul National University	0.09
	6	Institut National de la Sante et de la Recherche Medicale, Harvard Medical School, University of Texas System	32	University of Texas System	0.07
	7	University of London	30	University of Sydney, University of Manchester, University of Liverpool	0.06
	8	Chinese Academy of Sciences	27	Harvard Medical School, University of London, State University System of Florida, University of Oxford, Tufts University, etc.	0.05
	9	Universidate de Sao Paulo	26	Universite Paris Cite, Sungkyunkwan University, NSW Health, Johns Hopkins University, Sun Yat Sen University, etc.	0.04
	10	Consejo Superior de Investigaciones Cientificas, Mahidol University, Zhejiang University	25	Universidate de Sao Paulo, Consejo Superior de Investigaciones Cientificas, Mahidol University, Zhejiang University, King Saud University, etc.	0.03
Author	1	Benjakul Soottawat	18	Benjakul Soottawat	0.00
	2	Miot Helio Amante	17	Miot Helio Amante	0.00
	3	Sarkar Rashmi	14	Sarkar Rashmi	0.00
	4	Chang Sung Eun	12	Chang Sung Eun	0.00
	5	Roulin Alexandre	10	Roulin Alexandre	0.00

**FIGURE 4:**
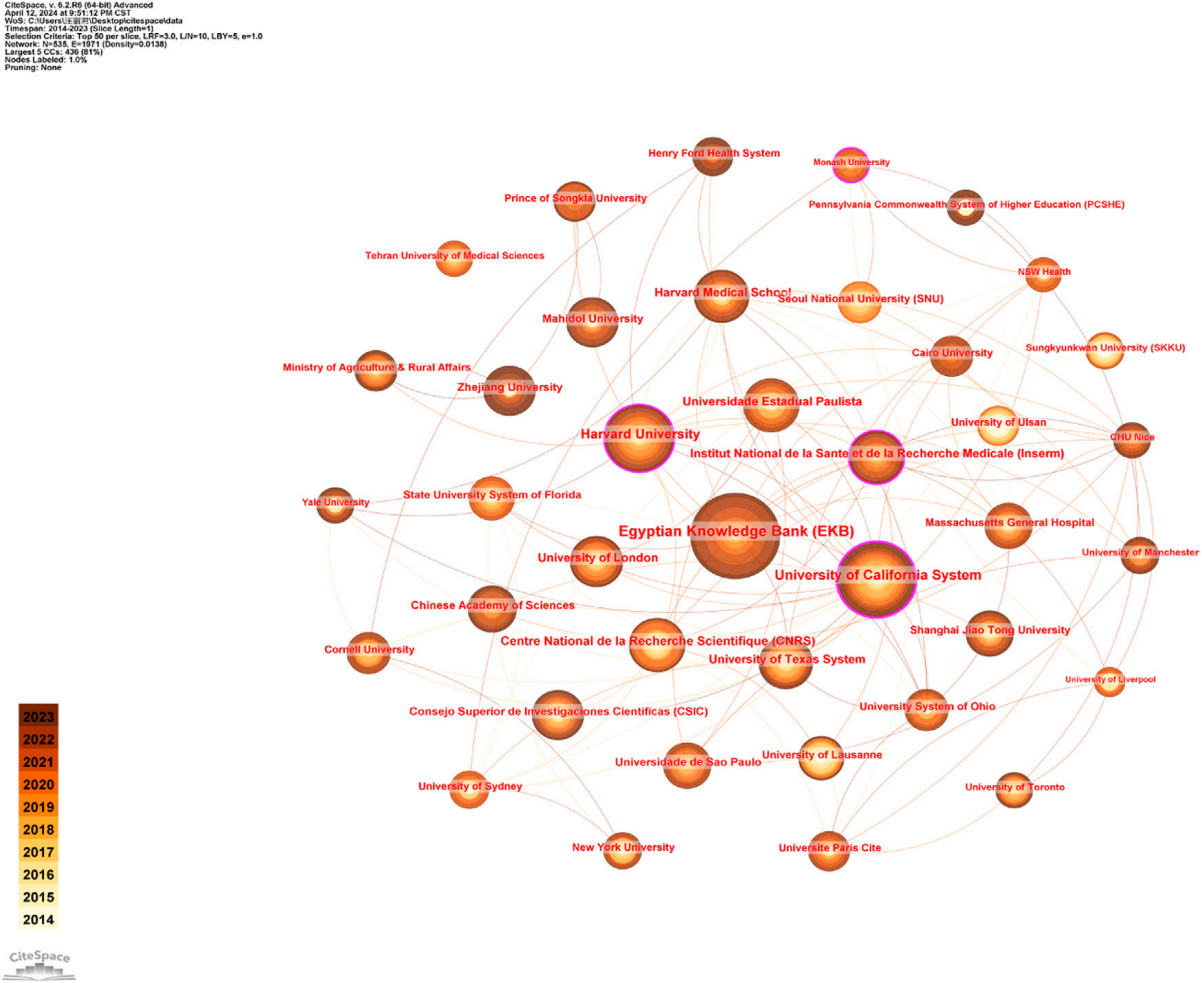
Institutions’ collaboration network of research on melasma.

A total of 535 organizations have published in this area. Table 1 indicates that the Egyptian Knowledge Bank (80) is the most prolific contributor to this field, followed by the University of California System (61) and Harvard University (50). The largest centrality value is for the University of California System, followed by Institut National de la Sante et de la Recherche Medicale and Monash University. Although Monash University has a centrality value of 0.11, it has only 11 publications. An analysis of Table 1 reveals that the Egyptian Knowledge Bank (publications: 80, centrality: 0.1), University of California System (publications: 61, centrality: 0.27), and Harvard University (publications: 50, centrality: 0.1) are leading institutions in the field. Figure 4 demonstrates a predominantly robust institutional collaboration within the field. Nonetheless, certain institutions, such as Tehran University of Medical Sciences and University of Lausanne, which have published more than 10 articles, exhibit limited or no collaboration with their peers.

**FIGURE 5:**
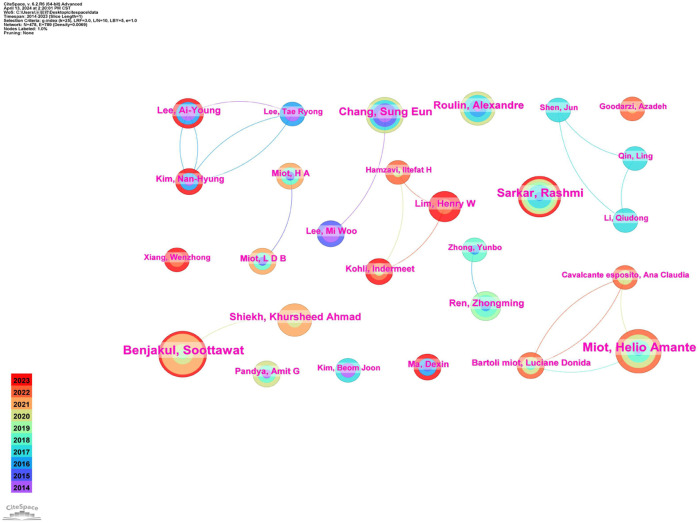
Author’s collaboration network of research on melasma.

A total of 478 authors have published papers related to Melanosis from 2014 to 2023. Table 1 lists all the authors with the top five number of published papers. According to the results analyzed by Citespace software, the centrality value of each author is 0.00. The authors who are in the top three in terms of the number of published articles are Benjakul Soottawat (18), Miot Helio Amante (17) and Sarkar Rashmi (14). The authors who have published more than five papers are shown in Figure 5. Benjakul Soottawat, which has the largest node, has had some cooperation with Shiekh Khursheed Ahmad only in the last few years. It is worth noting that Lim Henry W and Kohli Indermeet have worked more closely together in recent years.

## Research topic analysis

### Research basis: analysis of highly co-cited references

**FIGURE 6:**
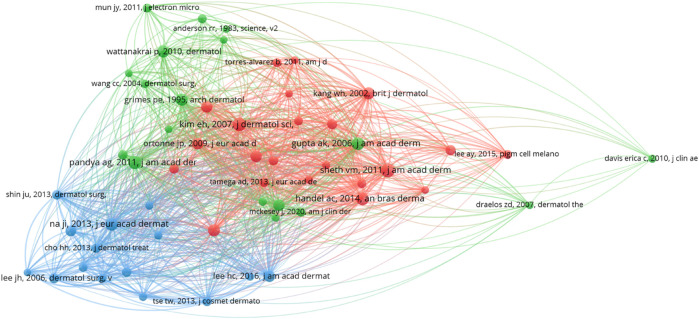
Visualization of clustering map of highly co-cited references.

Co-cited references are references that are cited jointly by the researcher. Co-cited literature, inherently connected to the original article, serves as a foundation for discerning the underlying research themes associated with melasma. We used VOSviewer to plot co-citation references, and the results showed 74,149 co-cited references were cited. When we set the minimum number of citations of a cited references to 40, there are 80 documents left that meet this threshold. We ultimately chose the largest collection of connected items (Figure 6).

The network diagram of highly co-cited literatures is divided into three clusters corresponding to the three colors in the figure. The red clusters are mainly related to the cutaneous vascular lesions of melasma (Kim et al., 2007), histopathology (Kang et al., 2002), melasma severity scores (Pandya et al., 2011), and the characteristic facial distribution of melasma in some regions (Tamega Ade et al., 2013). Green clusters are mainly studied with physical methods [Low-fluence Q-switched neodymium-doped yttrium aluminum garnet (1,064 nm) laser] for the treatment of melasma. It selectively modifies the three-dimensional structure of melanocytes and the ultrastructure of melanosomes (Mun et al., 2011), and selectively targets skin microvessels (Anderson and Parrish, 1983). Most of the literature in the blue cluster is on clinical trials or clinical observations of tranexamic acid for melasma. The top 10 most co-cited documents are shown in Table 2. We can find that basic research focuses on melanocytes and skin microvasculature, and clinical research focuses on laser therapy and drug therapy (tranexamic acid). Susana Clusella Trullas et al. (2007) published one of the most co-cited articles, which summarizes the morphological and molecular mechanisms of melanosis, arguing that the phenomenon of melanosis is compatible with low temperatures and is related to the behavior and physiology of the organism. Studies in the co-cited literature have demonstrated that the pathological basis and therapeutic modalities of melanosis are closely related to the skin microvasculature and melanocytes (melanosomes), and that the onset and progression of the disease process is influenced by temperature and individual behavioral physiology.

**TABLE 2:** The top 10 co-cited references and highly cited references.

Item	Ranking	Title	Doi	Citations
Co-cited references	1	Thermal melanism in ectotherms	10.1016/j.jtherbio.2007.01.013	165
	2	Melasma: a comprehensive update: part I	10.1016/j.jaad.2010.12.046	121
	3	The vascular characteristics of melasma	10.1016/j.jdermsci.2007.01.009	113
	4	Reliability assessment and validation of the Melasma Area and Severity Index (MASI) and a new modified MASI scoring method	10.1016/j.jaad.2009.10.051	109
	5	Melasma: a clinical and epidemiological review	10.1590/abd1806-4841.20143063	108
	6	Effect of tranexamic acid on melasma: a clinical trial with histological evaluation	10.1111/j.1468-3083.2012.04464.x	105
	7	The treatment of melasma: a review of clinical trials	10.1016/j.jaad.2006.02.009	102
	8	Melasma: histopathological characteristics in 56 Korean patients	10.1046/j.0007-0963.2001.04556.x	96
	9	Low-fluence Q-switched neodymium-doped yttrium aluminum garnet (1,064 nm) laser for the treatment of facial melasma in Asians	10.1111/j.1524-4725.2009.01383.x	95
	10	Melasma: an Up-to-Date Comprehensive Review	10.1007/s13555-017-0194-1	93
Highly cited references	1	Melasma: an Up-to-Date Comprehensive Review	10.1007/s13555-017-0194-1	72
	2	Melasma, a photoaging disorder	10.1111/pcmr.12684	67
	3	Oral tranexamic acid (TA) in the treatment of melasma: A retrospective analysis	10.1016/j.jaad.2016.03.001	50
	4	Melasma Treatment: An Evidence-Based Review	10.1007/s40257-019-00488-w	49
	5	A review of laser and light therapy in melasma	10.1016/j.ijwd.2017.01.004	46
	6	Oral Tranexamic Acid for the Treatment of Melasma: A Review	10.1097/DSS.0000000000001518	45
	7	Randomized, placebo-controlled, double-blind study of oral tranexamic acid in the treatment of moderate-to-severe melasma	10.1016/j.jaad.2017.09.053	43
	8	Effect of tranexamic acid on melasma: a clinical trial with histological evaluation	10.1111/j.1468-3083.2012.04464.x	42
	9	Melasma: a clinical and epidemiological review	10.1590/abd1806-4841.20143063	40
	10	Efficacy and possible mechanisms of topical tranexamic acid in melasma	10.1111/ced.12835	38

### Analysis of highly cited references

We analysed the highly cited literature through Citespace. Table 2 presents the top 10 highly cited literature related to melasma research. Highly cited literature generally has a high academic impact, and it can reflect the frontiers or hotspots of a particular field. Table 2 delineates the distribution of research topics, with six papers focusing on the use of tranexamic acid for melasma treatment and two examining laser therapy approaches. Only one of the 10 papers examined the underlying pathology behind the onset of melasma, which is characterized as a photodamaged dermatosis. It suggests that current research for melasma is centered around clinical studies or basic research on tranexamic acid.

### Analysis of co-occurring keywords, burst term and cluster analysis

**FIGURE 7:**
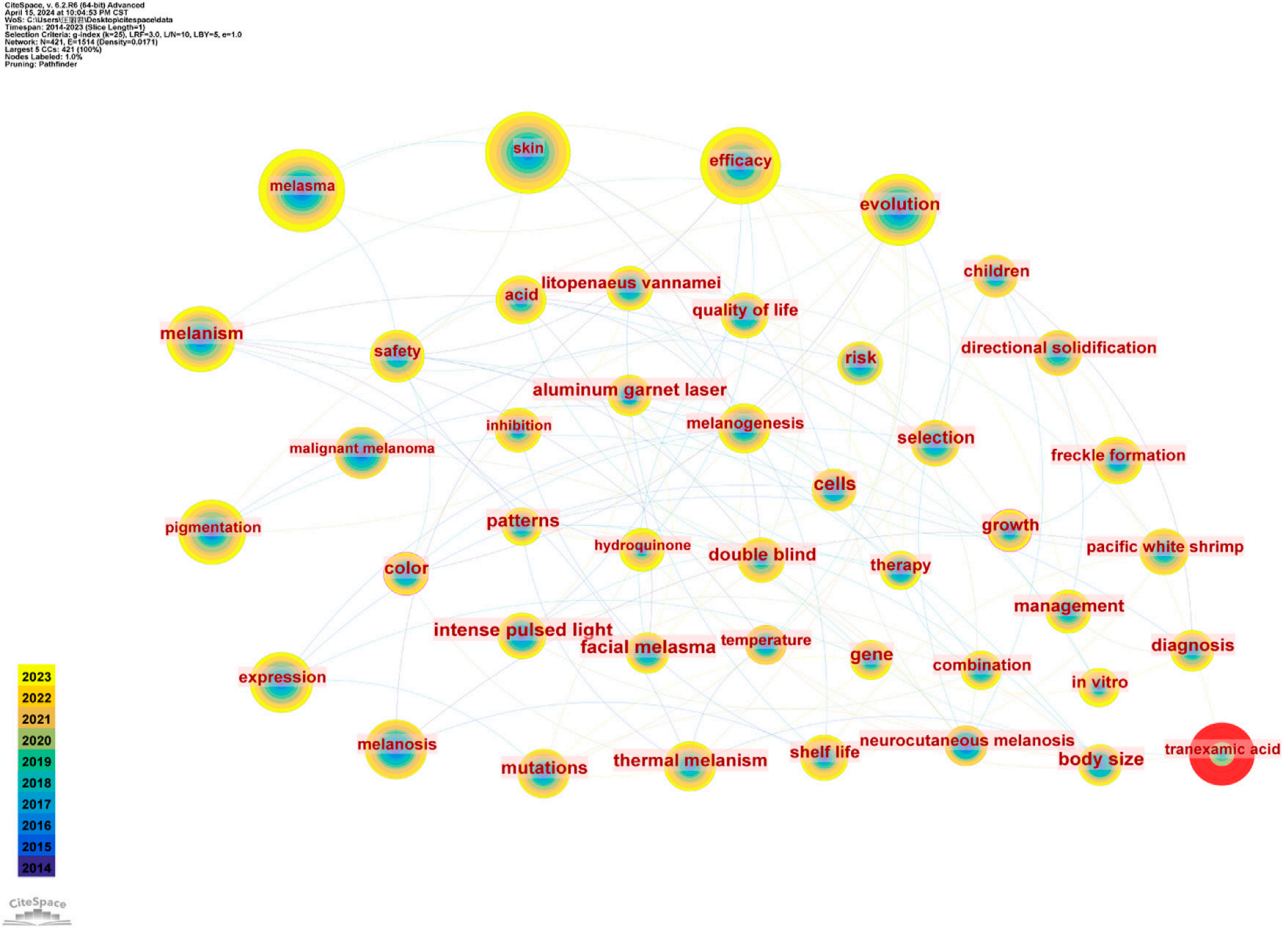
Co-occurring keywords map.

The analysis of keyword co-occurrence serves as a valuable tool for discerning research trends within a field and for identifying potential focal points for future inquiry. We conducted a frequency and centrality analysis of keywords associated with melanosis from 2014 to 2023, as presented in Table 3, identifying the top 20 most prominent keywords. In the context of melasma research, “melasma” emerges as the predominant term, succeeded by “skin,” “efficacy,” “evolution,” “melanism,” “pigmentation,” “tranexamic acid,” “expression” among others. It can be inferred that the hotspots of the study are mainly focused on clinical treatment and clinical performance. It should be noted that these keywords also incorporate research on other aspects of melasma. In addition, “malignant melanoma,” “thermal melanism,” and “melanogenesis” all appear more than 75 times. The greater the centrality value, the greater the bridging role of the keyword in the field of study. As shown in Table 3, “color” is at the center of the field, the remaining keywords include “cells,” “antioxidant,” “postinflammatory hyperpigmentation,” “mechanism,” “skin pigmentation” and so on. The keyword co-occurrence network diagram is shown in Figure 7. The higher the frequency, the hotter it gets. The connection between the keywords indicates that they are studied together.

**TABLE 3:** Top 20 keywords in terms of count and centrality.

Ranking	Keyword	Count	Keyword	Centrality
1	Melasma	211	Color	0.13
2	Skin	208	Growth	0.11
3	Efficacy	179	Cells	0.1
4	Evolution	158	Antioxidant	0.09
5	Melanism	145	Lesions	0.08
6	Pigmentation	133	Patterns	0.08
7	Tranexamic acid	124	Association	0.08
8	Expression	117	Postinflammatory hyperpigmentation	0.07
9	Melanosis	113	Mechanism	0.07
10	Safety	90	Facial melasma	0.06
11	Malignant melanoma	87	Melanoma	0.06
12	Thermal melanism	82	Skin pigmentation	0.06
13	Mutations	78	Activation	0.06
14	Melanogenesis	76	Identification	0.06
15	Acid	75	Freckles	0.06
16	Selection	71	Differentiation	0.06
17	Intense pulsed light	70	Behavior	0.06
18	Freckle formation	69	Population	0.05
19	Shelf life	68	Melanosis	0.05
20	Pacific white shrimp	68	Acid	0.05

**FIGURE 8:**
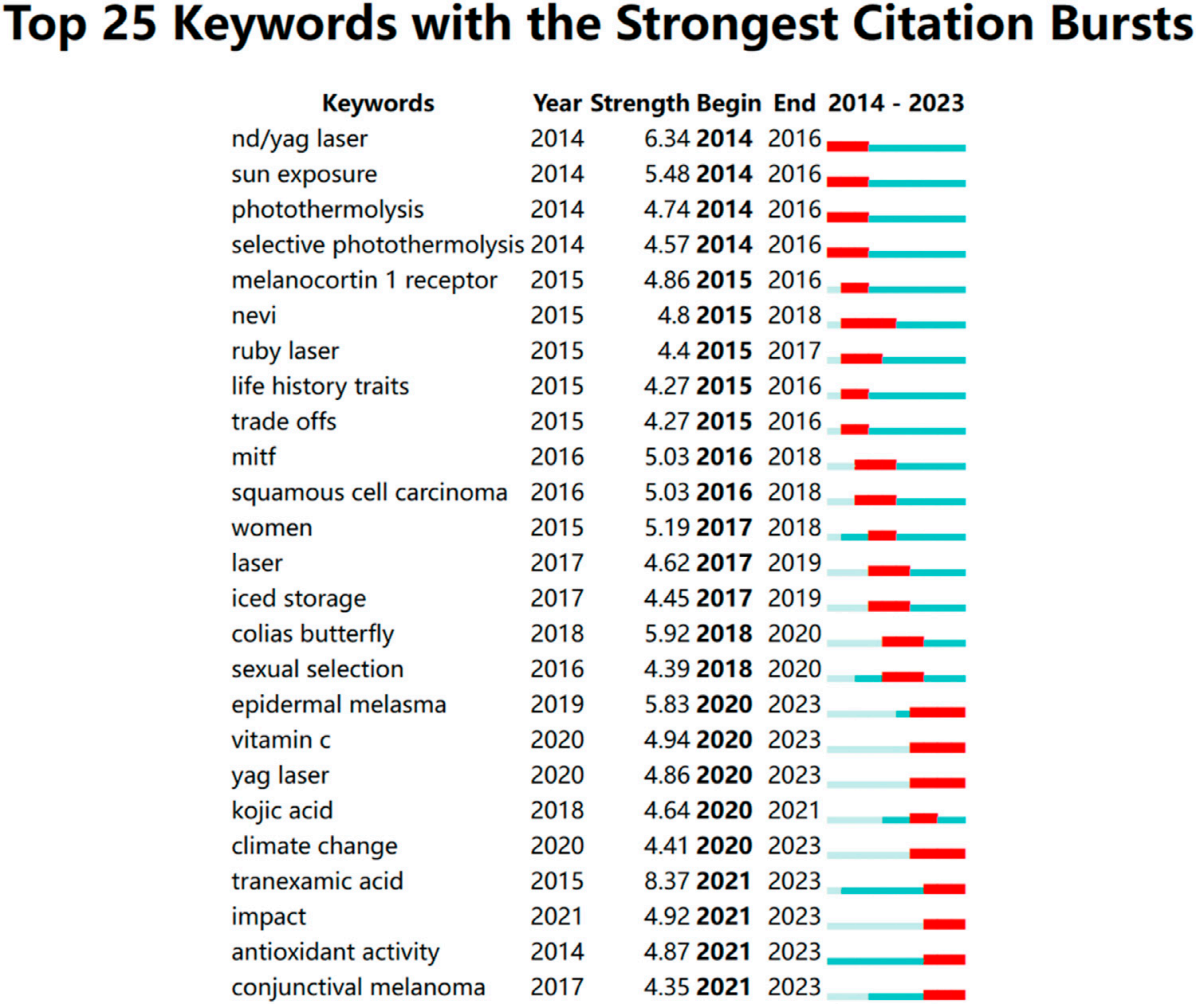
Top 25 keywords with the strongest citation bursts.

A keyword burst is a sudden increase in research content at a given moment. Studying keyword bursts can predict potential research trends in that research area. Figure 8 shows the 25 keywords with the strongest burst intensity in this research area. The blue line indicates the time interval and the red line indicates the duration of the outbreak for that keyword. We can see that the keyword burst has evolved from “nd/yag laser,” “sun exposure,” “photothermolysis,” “melanocortin 1 receptor,” to the current dimensional change of “vitamin C,” “yag laser,” “tranexamic acid,” “antioxidant activity.” This suggests that research on melasma is at a therapeutic level in recent years.

Keyword clustering allows you to classify and categorize research topics and understand the different research directions in the research field. In this study, Citespace was used to cluster melasma-related keywords from 2014 to 2023. There are eight clusters, the modularity is Q = 0.5936 (>0.3), and the weighted mean silhouette is S = 0.8545(>0.7). These metrics indicate that the clustering is well-founded. The specific clustering clusters are shown in Table 4 (Remove a cluster with a Silhouette value of 0). This indicates that there is a lot of research on the treatment of melasma and the observation of its efficacy. Melasma has always been a common but extremely difficult disease to treat. Current research primarily focuses on mitigating hyperpigmentation, as well as the prevention and treatment of melasma. The concept of “melanogenesis” serves as a pivotal link between mechanistic and clinical research endeavors.

**TABLE 4:** The top eight keyword clustering.

Cluster	Size	Silhouette	Mean (Year)	Top Terms(LSI)
#0 melasma	99	0.854	2016	Melasma; efficacy; postinflammatory hyperpigmentation; safety; therapy | tranexamic acid; low fluence; post inflammatory hyperpigmentation; postinflammatory hypermelanosis; anti-androgenic agents
#1 melanism	73	0.899	2016	Thermal melanism; glogers rule; ultraviolet-b radiation; vertical stratification; uv-b radiation | colour polymorphism; sexual dimorphism; antagonistic selection; female ornament; batesian mimicry
#2 neurofibromatosis type 1	66	0.825	2016	Neurofibromatosis type; au-lait spots; von recklinghausen disease; orthopaedic manifestations; mucosal melanomas | neurocutaneous melanosis; kinky hair syndrome; neurocutaneous syndromes; ataxia telangiectasia; central nervous system
#3 pacific white shrimp	54	0.874	2018	Pacific white shrimp; inhibition kinetic; shrimp freshness; acidic electrolyzed water; microstructure | litopenaeus vannamei; inhibition; microstructure; inactivation; collagen
#4 melanogenesis	51	0.829	2017	Melasma; hydroquinone; neutral red; *in vitro*; deoxyarbutin | skin pigmentation; protein carbonylation; transcription factor; camp response element; butyroside d
#5 directional solidification	38	0.937	2017	Directional solidification; varying cross section; surface defect; superalloy; focused ion beam | freckle formation; model; transport phenomena; microgravity; alloys
#6 skin cancer	34	0.743	2016	Skin cancer; acne vulgaris; neurofibromatosis type; sentinel lymph node biopsy; desmoplastic melanoma | risk factors; primary prevention; melanocytic nevi; malignant melanoma; public health surveillance
#7 atrophic acne scars	5	0.951	2017	Percutaneous collagen; atrophic acne; photodynamic therapy; laser therapy; oral pigmentation | ocular surface; ocular tumours; anti-cancer agents; squamous neoplasia; choroidal melanoma

## Discussion

### Publication trend analysis

From 2014 to 2023, the amount of literature on melasma is generally increasing, suggesting that this area remains a research hotspot.

### Cooperative relationship

In terms of countries/regions, the top three countries with the highest number of published articles are the United States, China, and South Korea, in that order. Among them, the United States has the largest centrality value of 0.4, indicating that it has a relatively close cooperation with other countries/regions. Although China holds the second position in publication count, its centrality value is a modest 0.06. Comparatively, South Korea, ranking third, exhibits an even lower centrality value of 0.04. Research on melasma in China, and by extension, Asia, could potentially foster enhanced collaboration with international counterparts. In terms of institutional collaboration, the University of California System has the highest centrality value and a high number of publications, indicating the important role of the United States in melasma research. For author collaboration, the centrality values are all 0, indicating that there is not a strong collaboration between authors in the field of melasma research.

### Research basis and hot spot

We use bibliometric analysis to help scholars grasp the trends in this research area. According to the keyword burst analysis, we can see that scholars’ understanding of melasma has gradually deepened, and “how to effectively treat melasma” is the current research hotspot. In addition, we analyzed the basis and frontiers of research on melasma based on co-cited and highly cited literature. Research has focused on the role of skin microvasculature, melanocytes in melasma. The number of skin microvessels as well as vascular endothelial growth factor (VEGF) are elevated in the lesional skin of melasma patients (Kim et al., 2007). Abnormal activation of melanocytes and accumulation of melanin and melanosomes directly contribute to skin darkening (Artzi et al., 2021). In conclusion, the study of the effects of skin vasculature and melanocytes on melasma formed the basis for subsequent research.

### Skin microangiopathy is one of the triggers of melasma

It has been demonstrated that the blood vessels at the site of melasma lesions are diseased (Kim et al., 2007). In the dermis of skin lesions, there is an abnormal aggregation of VEGF and mast cell-derived factors secreted by dermal blood vessels (Kwon et al., 2016). In melasma patients, a more pronounced capillary correlates with a heightened expression of PAR-2, particularly within the spinous and basal layers keratinocytes interface with melanocytes (Lee et al., 2017). In contrast to the PBS-injected mice, the skin of the VEGF-injected mice exhibited a significant increase in the mRNA level of PAR-2 (Lee et al., 2017). VEGF is a key factor in normal and pathologic angiogenesis (Melincovici et al., 2018). Niti Khunger and colleagues have demonstrated through experimental studies that the expression of VEGF is present in melasma lesions; however, this does not correlate with an in the demarcation of facial pigmentation (Khunger et al., 2020). Anti-VEGF drugs have been widely utilized in the treatment of diseases associated with retinal vasculopathy (Porta and Striglia, 2020).

Based on the expression characteristics of VEGF in melasma lesions, a hypothesis has been proposed to use vascular endothelial growth factor inhibitors in combination with anti-estrogenic drugs for topical administration in the treatment of melasma (Cohen, 2017).

### Melanocytes are responsible for the formation of dark spots on the skin

Melanocytes have been important in melasma research. Inappropriate activation of melanocytes and aggregation of melanin and melanosomes are important pathologic mechanisms in the development of melasma (Artzi et al., 2021). As a result, most treatments for melasma are centered around reducing melanin and inhibiting melanin formation (Cassiano et al., 2022).

Research has demonstrated that there are racial differences in epidermal melanin content and melanosome dispersion (Taylor, 2002). The distribution of melanin in human skin is heterogeneous, and normal and pathologic skin pigmentation are also highly heterogeneous (Vicente et al., 2022). Melanin in the skin itself has photoprotective properties (Ortonne, 2002), but when skin homeostasis is disrupted, melanin may accumulate abnormally leading to facial dark spots. The molecular mechanisms behind this abnormal skin pigmentation are related to the cytosolic emesis of melanin by melanocytes and the transfer of melanin into the surrounding keratin-forming cells for internalization, processing, and polarization (Bento-Lopes et al., 2023). By comparing the ultrastructural features of UV-induced senescent melanocytes *in vitro* with those of hypopigmented senescent skin *in vivo*, foreign scholars have demonstrated that senescent melanocytes are characterized by impaired melanosome transport leading to melanin accumulation, and that glycolytic metabolism facilitates this process, as demonstrated by the results of single-cell transcriptome analysis (Park et al., 2023).

### Tranexamic acid and lasers are important treatments for melasma

Tranexamic acid is a protease inhibitor that interferes with melanin production on the front end and prevents the transit and spread of already produced melanin on the back end. A clinical study reported significant improvement in melasma lesions in 85% of patients following a 4-week oral administration of compound tranexamic acid, with the figure rising to 100% after a 16-week treatment period (Li et al., 2014). However, more research is needed on oral tranexamic acid for melasma to prove its safety and effectiveness for long-term use (McKesey et al., 2020). Topical tranexamic acid significantly reduced melanin content in the epidermis and showed a downward trend in the expression of vascular endothelial factor (Kim et al., 2016).However, achieving therapeutic concentrations of topical tranexamic acid in the skin presents a challenge, despite the potential for adjuvant enhancers to facilitate temporary attainment of these levels (Ng et al., 2020). Therefore, it is critical to improve the delivery of topical tranexamic acid. Currently, nanotechnology has been maturely used in various types of topical hypopigmentation agents; compared with traditional topical drugs, topical hypopigmentation agents combined with nanocarriers can realize the advantages of efficacy at low concentration, targeting different skin layers, and rapid onset of action (Hatem et al., 2020).

Melasma patients exhibit melanocytes in an active state, which are highly sensitive to stimuli that may exacerbate pigmentation and result in repigmentation (Liu et al., 2023). It has been documented that there is a risk of persistent recurrence or post-inflammatory hyperpigmentation with the use of Q-tuned lasers for melasma (Passeron, 2012). Therefore, the choice of laser is particularly important. At present, the Q-modulated laser with large spot, low energy and long wavelength is still recommended internationally as the preferred pigmented laser for treating melasma. Zheng H and other scholars treated patients with freckles with melasma using the Q-modulated 1,064 nm laser, with a 100% freckle remission rate and a 39.4% melasma remission rate (Zheng et al., 2023). Hong JK and other scholars used 785 nm picosecond laser to treat hyperpigmentation disease in Asians, and the pigmentation was improved (Hong et al., 2022a). However, from domestic and international experience, picosecond laser has not been found to be superior to Q-tuned laser in treating melasma (Hong et al., 2022b).

Clinical studies have found that YAG laser-assisted delivery of tranexamic acid is suitable for patients who are not sensitive to conventional treatment methods, and that the combination of oral TA can enhance the therapeutic effect of this regimen (Botsali et al., 2022). Through systematic review and meta-analysis, Qaisar Ali Khan and other scholars concluded that laser treatment combined with topical tranexamic acid significantly reduced melasma area of severity index (MASI) (Khan et al., 2023).

### Development of natural antioxidant drugs is a research trend

Oxidative stress plays a role in the progression of melasma disease. A study by Shweta Katiyar et al. (2024) found that the basal levels of systemic antioxidants were lower in patients with melasma than in healthy individuals, suggesting that increased oxidative stress may affect tyrosinase activity through the anabolic pathway of melanin synthesis. Thus the use of antioxidants may help in the treatment of melasma.

A clinical study found that lesions treated with nanosomal vitamin C showed better results than those treated with glycolic acid (Sobhi and Sobhi, 2012). A cream containing Licorice, Belides, Emblica is therapeutically safer than a cream containing hydroquinone (Costa et al., 2010). Japanese scholars found that grape seeds are rich in a powerful antioxidant - proanthocyanidins, which can effectively reduce the melanin index in the skin lesions, reduce hyperpigmentation, and long-term use of no obvious side effects, the efficacy of safe and effective (Yamakoshi et al., 2004). Intake of red orange extract enhances the antioxidant capacity of the skin and improves hyperpigmentation (Puglia et al., 2014). Yan Yi Sim and colleagues found that purified kenaf leaves extracts (PKLE) and kenaf seed oil (KSO) from Hibiscus cannabinus L. leaf and seed inhibited tyrosinase activity in normal human dermal fibroblasts and epidermal melanocytes activity (Sim et al., 2022). We summarize some of the natural products with antioxidant and melanin inhibiting properties in Table 5.

**TABLE 5:** Summary of natural products for the treatment of melasma in models.

Natural product name	Active ingredient/main ingredient	Models	Range of dosage	Targets/pathway/process/mechanism	References
Phyllostachys pubescens	Bamboo peel extract (Betulinic acid, tachioside, and 1,2-dilinolenin)	B16 melanoma cells(mice) three-dimensional human skin model	0.1%, 0.2%, 0.25%, 0.3%, 0.4%, 0.5%, 2, 5%	melanin, TYR	Ashour et al. (2022)
Vitis vinifera Leaf	Vitis vinifera Leaf Extracts (gallic acid, chlorogenic acid, epicatechin, rutin, resveratrol)	*In silico* (no cell)	3.84 mg/mL	TYR	Lin et al. (2017)
Purple Glutinous Rice (Oryza sativa L.cv.Pieisu 1 CMU)	PES1CMU extracts (PES1CMU-DFRB, PES1CMU-H)	IBMX-stimulated B16 melanoma cells (mice skin cell) H2O2-induced human fibroblast cell	0.01, 0.1 mg/mL	mushroom TYR, melanin, TYR, malondialdehyde, MMP-2	Linsaenkart et al. (2023)
Leaves of Prunus persica(L.) Batsch var.Florida Prince	PPEE (kaempferol 3-O--4C1-(6-O-3,4-dihydroxyphenylacetyl glucopyranoside))	Human Keratinocytes	0–0.5 mg/mL	TYR	Mostafa et al. (2021)
Pomegranate	Pomanox	UV-induced human fibroblast Hs68 cells	394.7 μg/mL	TYR	Mariné-Casadó et al. (2023)
Clove	Ellagitannin casuarictin	B16-F10 (mice cell)	30 µM	TYR, mushroom TYR, MITF	Goenka et al. (2021)
European Propolises	Propolis Extracts (phenolic acid glycerides, galangin, chrysin, pinocembrin, and pinobanksins-3-O-acetate)	In silico (no cell)	25, 50, 100 μg/mL	mushroom TYR,TYR	Widelski et al. (2022)
Elaeagnus umbellata	EU extract and fractions(luteolin)	α-MSH -induced B16-F10 melanoma cells	12.5, 25, 50 μg/mL	TYR, CREB, ERK, MITF, TRP-1, TRP-2	Lee et al. (2021)
Milk	MHIR	Mel-Ab (mice cell) α-MSH-induced B16-F10 melanoma cells	5 μM, 50 μM, 100 μM	MC1R, CREB	Kong et al. (2020)
Olive leaf	oleuropein (OP), oleocanthal (OL), oleacein (OC)	B16-F10 (mice cell) Epidermal Human Melanocytes	5 μm, 200 nM	MITF, mTOR, MAPK, JAK-STAT, KEGG, p53, cAMP, PI3K-Akt, Wnt, MC1R, Kit, Gsk3β, TYR, TYRP1	Cho et al. (2024)
Melissa officinalis	MOE, UMOE	B16-F1 (mice cell)	200 μg/mL	TYR, TRP-1, TRP-2	Jun et al. (2011)
Potentilla paradoxa Nutt	PP-EE(α-Linolenic acid)	α-MSH -induced B16-F10 (mice cell)	50, 100 μg/mL	MITF, PKA, TYRP1, TYRP2	Lee et al. (2022)
Deschampsia antarctica	EDA	Artificial blue light-induced melanocytes	0.1 mg/mL	P38	Lorrio et al. (2020)
Mutated shiitake	the total phenolic content (TPC) and total flavonoid content (TFC)	the melanocytes of zebrafish human foreskin fibroblast (HS27)	100,300 μg/mL; 679 ± 19.7 μg/mL	melanin, pGSK3β	Mahmood et al. (2021)
Camellia japonica seeds	CJS-EO (hexamethylcyclotrisiloxane)	B16F10 (mice cell)	31.25–500 ppm	TYR, melanin, TRP-1, TRP-2	Ha et al. (2021)
Soybean	SCE (the total phenolic content)	α-MSH -induced B16-F10 (mice cell)	1 mg/mL	TYR, melanin, TRP1, TRP2, MITF	Bodurlar and Caliskan (2022)
Citrus cultivars peels	β-elemene, farnesene, limonene	α-Melanocyte-stimulated B16BL6 cells.	10^–7^–10^−6^%	TYR, melanin, MITF, MC1R	Yang et al. (2023)
Fomitopsis castanea mycelia	FEPS(Exopolysaccharide)	α-MSH -induced SK-MEL-5 human melanoma cells	16.5 mg/mL; 50, 100 μg/mL	mushroom TYR, TYR	Jin et al. (2019)
Monascus purpureus	monascuspirolide B (7), Ergosterol Peroxide (8)	α-MSH -induced B16-F10 (mice cell)	5–20 μM	TYR, melanin	Wu et al. (2021)
Juglans mandshurica	2-[4-(3-hydroxypropyl)−2-methoxyphenoxy]−1,3-propanediol	B16F10 melanoma cells primary human epidermal melanocytes (PHEMs)	0.5, 1 μM; 10 μM	melanin, MITF, p-ERK1/2、ERK1/2, TYR, p-CREB	(Kim J. Y. et al., 2019)
Prunus mahaleb L.	2-O-β-glucopyranosyloxy-4-methoxy-hydrocinnamic acid, Dihydromelilotoside,2-O-β-glucosyloxy-4-methoxy trans-cinnamic acid	α-MSH-stimulated B16F10 melanoma cells	20–400 μg/mL	mushroom TYR, TYR,melanin	Bayrakçeken Güven et al. (2023)
Helichrysum italicum	essential oils (α-pinene, α-muurolene)	*In silico* (no cell)	1 mg/mL	TYR	Nebrigić et al. (2023)
Fungi	3,4-Dihydroxybenzalacetone	B16-F10 (mice cell) human epidermal melanocytes (HEMs). T	2.5–10 μM	TYR, TRP-1, TRP-2, MITF, cAMP/PKA, AKT/GSK3β, MEK/ERK, PMEL17	Liu et al. (2021)
Withania somnifera(WS), Solanum nigrum (SN)	WSEA (high gallic acid, apigenin, kaempferol), SNEA (high caffeic acid, gallic acid, kaempferol, quercetin)	*In silico* (no cell)	1 mg/mL	TYR	Hameed and Akhtar (2018)
C. fistula flower	C. fistula flower extract (Vanillic acid, Protocatechuic acid, Gallic acid, Coumaric acid, Ferulic acid, Chlorogenic acid, Catechins)	human skin fibroblasts *In silico* (no cell)	50–200 μg/mL	MMP-2; TYR	Limtrakul et al. (2016)
Streblus taxoides Wood	ω-Hydroxymoracin C, moracin M, moracin C, 3, 4, 3′, 5′-tetrahydroxybibenzyl, piceatannol	B16-F1 melanoma cells	25,50 μg/mL	TYR, melanin, TRP1、TRP2, MITF	Parndaeng et al. (2023)
Catalpa ovata	COE [the ethyl acetate fraction (EF)]	B16-F1 melanoma cells	25, 50, 100 μg/mL	TYR, melanin, TRP1, MITF, p38, JNK, ERK, MAPK, p-CREB, cAMP,	Kim et al. (2023)
Bee pollen of Helianthus annuus L.	Sunflower bee pollen (SBP) extract (Safflospermidine A and B)	*In silico* (no cell)	50 μg/mL	TYR	Khongkarat et al. (2020)

Natural antioxidants have great potential to improve pigmentation and treat melasma. Improving the delivery and stability of natural antioxidants is key to improving efficacy. For example, the biggest challenge in utilising vitamin C is maintaining its stability and improving its delivery to the active site (Caritá et al., 2020). It has been demonstrated that promoting transdermal penetration of stem cell-derived exosomes can effectively improve the efficacy of treating melasma (Wang et al., 2023). Improving the delivery mode of existing natural antioxidants and discovering new natural antioxidants may become the new wind in the field of melasma research.

### Understanding climate change promises new breakthroughs in Melasma course management

Melasma formation is a complex process, and the literature proposes that not only skin blood vessels and melanocytes are involved, but also senescent dermal fibroblasts, which secrete VEGF to directly stimulate neovascularization, and these newborn endothelial cells release endothelin-1, which upregulates melanogenic pathways in melanocytes (Regazzetti et al., 2015). Even mast cell-mediated inflammatory responses are included (Phansuk et al., 2022). Therefore, the timely removal of senescent cells to maintain skin homeostasis is also crucial in the management of melasma (Kim M. et al., 2019).Melasma formation is influenced by a number of factors. Therefore, it is particularly important to optimize the management of melasma over the course of the disease.

In a study of *Sciurus carolinensis*, it was found that the animal’s melanism was weakest in cities with warmer winter temperatures (Cosentino and Gibbs, 2022). Scholars in dermatology-related specialties have suggested in a paper that climate change interacts with skin health and that understanding climate change is critical to the proper management of refractory skin conditions (Belzer and Parker, 2023).

## Limitations

Firstly, the WOSCC database was selected for our study and only English articles were searched, which may result in some missing data from the literature. Second, some authors or organisations have different name formats in the WOSCC database, perhaps implying that their research counts may be scattered. Finally, due to publication bias, all conclusions in this study were drawn from published studies, but there may be literature that will never be published, so there may be publication bias. However, the results of our analyses are sufficient to accurately describe the current status and trends in global research on melasma.

## Conclusion

This is the first study to analyse melasma using bibliometric and visualisation methods, demonstrating the overall state of research in this area of study and exploring future research directions. The United States, China, and South Korea are the regions with the most publications. Currently, the main research hotspots related to melasma focus on treatment options, such as vitamin C, tranexamic acid, and lasers. Research trends may be natural antioxidants, science-based disease management.

In summary, melasma is a condition that is complex to treat and prone to recurrence requiring long-term treatment. Currently, melasma lacks a single effective treatment. However, we need appropriately designed clinical trials to further understand the efficacy of natural products on melasma in order to increase the choice of treatment modalities. In the future, the search for natural antioxidant drugs with high efficacy and low toxicity may be a potential research trend, based on existing treatments. And the development of a scientific disease management programme for melasma in different regions, climates and populations is the key to preventing the disease and reducing recurrence.
